# Crystal structure of [propane-1,3-diylbis(piperidine-4,1-di­yl)]bis­[(pyridin-4-yl)methanone]–4,4′-oxydi­benzoic acid (1/1)

**DOI:** 10.1107/S160053681401811X

**Published:** 2014-08-16

**Authors:** Emily M. Low, Robert L. LaDuca

**Affiliations:** a Heritage High School, Rogers, AR 72756, USA; b Lyman Briggs College, Department of Chemistry, E-30 Holmes Hall, 919 East Shaw Lane, Michigan State University, East Lansing, MI 48825, USA

**Keywords:** crystal structure, co-crystal, hydrogen bonding, piperazine-1,4-diylbis(pyridin-4-yl­methanone), 4,4′-oxydi­benzoic acid

## Abstract

In the title co-crystal, C_25_H_32_N_4_O_2_·C_14_H_10_O_5_, mol­ecules are connected into supra­molecular chains aligned along [102] by O—H⋯N hydrogen bonding. These aggregate into supra­molecular layers oriented parallel to (20-1) by C—H⋯O inter­actions. These layers then stack in an *ABAB* pattern along the *c* crystal direction to give the full three-dimensional crystal structure. The central chain in the dipyridylamide has an *anti*–*anti* conformation. The dihedral angle between the aromatic ring planes is 29.96 (3)°. Disorder is noted in some of the residues in the structure and this is manifested in two coplanar dispositions of one statistically disordered carb­oxy­lic acid group.

## Related literature   

For the preparation of piperazine-1,4-diylbis(pyridin-4-yl­methanone), see: Hou *et al.* (2003 ▶). For the preparation of divalent metal 4,4′-oxydibenzoate coordination polymers, see: Yang *et al.* (2009 ▶).
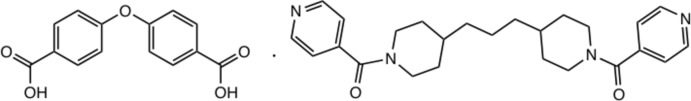


## Experimental   

### Crystal data  

C_25_H_32_N_4_O_2_·C_14_H_10_O_5_
*M* *_r_* = 678.77
Monoclinic, 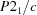

*a* = 16.032 (4) Å
*b* = 12.605 (4) Å
*c* = 17.259 (5) Å
β = 99.033 (7)°
*V* = 3444.4 (16) Å^3^
*Z* = 4
Mo *K*α radiation
μ = 0.09 mm^−1^
*T* = 173 K
0.22 × 0.17 × 0.15 mm

### Data collection  

Bruker APEXII CCD diffractometer
Absorption correction: multi-scan (*SADABS2012*; Bruker, 2012 ▶) *T*_min_ = 0.715, *T*_max_ = 0.745
55666 measured reflections
6304 independent reflections
3501 reflections with *I* > 2σ(*I*)
*R*_int_ = 0.095

### Refinement  

*R*[*F* ^2^ > 2σ(*F* ^2^)] = 0.055
*wR*(*F* ^2^) = 0.153
*S* = 1.03
6304 reflections
457 parameters
2 restraints
H atoms treated by a mixture of independent and constrained refinement
Δρ_max_ = 0.50 e Å^−3^
Δρ_min_ = −0.54 e Å^−3^

### 

Data collection: *APEX2* (Bruker, 2012 ▶); cell refinement: *APEX2*; data reduction: *SAINT* (Bruker, 2012 ▶); program(s) used to solve structure: *SHELXS97* (Sheldrick, 2008 ▶); program(s) used to refine structure: *SHELXL97* (Sheldrick, 2008 ▶); molecular graphics: *Crystal­Maker* (Palmer, 2007 ▶); software used to prepare material for publication: *OLEX2* (Dolomanov *et al.*, 2009 ▶).

## Supplementary Material

Crystal structure: contains datablock(s) I, pub. DOI: 10.1107/S160053681401811X/tk5333sup1.cif

Structure factors: contains datablock(s) I. DOI: 10.1107/S160053681401811X/tk5333Isup2.hkl

Supporting information file. DOI: 10.1107/S160053681401811X/tk5333Isup3.cml

**Fig. 1.**
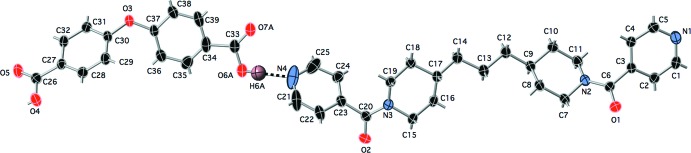
The formula unit of the title compound, showing 50% probability ellipsoids and atom numbering scheme. Most hydrogen atom positions are shown as grey sticks. Color codes: Red O, light blue N, black C, pink H. Only one of the disordered carboxylic acid groups is shown.

. DOI: 10.1107/S160053681401811X/tk5333fig1.tif
The formula unit of the title compound, showing 50% probability ellipsoids and atom numbering scheme. Most hydrogen atom positions are shown as grey sticks. Color codes: Red O, light blue N, black C, pink H. Only one of the disordered carboxylic acid groups is shown.

**Fig. 2.**
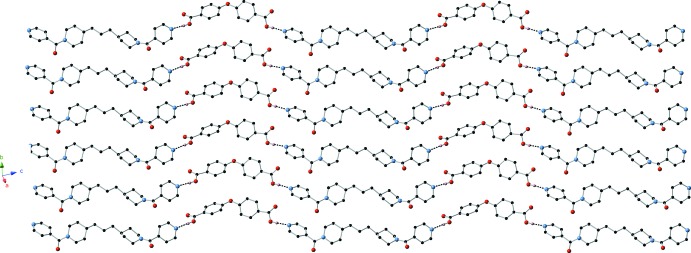
Layer pattern within the title compound constructed from supramolecular chains connected by O—H···N hydrogen bonding.

. DOI: 10.1107/S160053681401811X/tk5333fig2.tif
Layer pattern within the title compound constructed from supra­molecular chains connected by O—H⋯N hydrogen bonding.

**Fig. 3.**
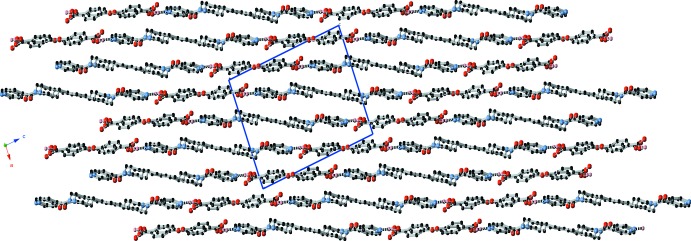
Stacking of supramolecular layers within the title compound.

. DOI: 10.1107/S160053681401811X/tk5333fig3.tif
Stacking of supra­molecular layers within the title compound.

CCDC reference: 1018294

Additional supporting information:  crystallographic information; 3D view; checkCIF report

## Figures and Tables

**Table 1:** Hydrogen-bond geometry (Å, °)

*D*—H⋯*A*	*D*—H	H⋯*A*	*D*⋯*A*	*D*—H⋯*A*
O4—H4*A*⋯N1^i^	0.84	1.83	2.665 (3)	176
O7—H7⋯N4	0.86 (2)	2.01 (3)	2.859 (7)	167 (8)
O6*A*—H6*A*⋯N4	0.86 (2)	1.79 (2)	2.652 (7)	177 (7)
C1—H1⋯O7*A* ^ii^	0.95	2.27	3.114 (6)	147
C12—H12*B*⋯O2^iii^	0.99	2.60	3.453 (4)	144
C19—H19*B*⋯O6^iii^	0.99	2.47	3.326 (6)	144

Symmetry codes: (i) 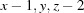; (ii) 
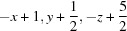
; (iii) 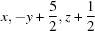.

## supplementary crystallographic information

### S1. Chemical context

Some divalent metal 4,4'-oxydibenzoate (oba) coordination polymers show intriguing self-penetrated topologies (Yang *et al.*, 2009). We therefore attempted to expand the scope of these materials by using the very-long spanning di­pyridyl ligand propane-1,3-diylbis(piperidine-4,1-diyl))bis­(pyridin-4-yl­methanone (ppbp). The title compound was obtained as colorless crystals through the hydro­thermal reaction of zinc nitrate, H_2_oba, and ppbp.

### S2. Structural commentary

The asymmetric unit of the title compound contains a full H_2_oba molecule with one of its carb­oxy­lic acid termini disordered in a 50/50 ratio, and a full ppbp molecule (Fig. 1). The H_2_oba and ppbp molecules are connected into supra­molecular chains (Fig. 2) aligned along [1 0 2] by O—H···N hydrogen bonding (Table 1) between unprotonated ppbp pyridyl N atoms and protonated H_2_oba carboxyl­ate O atoms.

### S3. Supra­molecular features

These chains aggregate into supra­molecular layers oriented parallel to the (2 0 1) crystal planes by C—H···O inter­actions between ppbp pyridyl C atoms and unprotonated H_2_oba carboxyl­ate O atoms (C···O distance = 3.114 (8) Å). These layers then stack in an *ABAB* pattern along the *c* crystal direction to give the full three-dimensional crystal structure of the title co-crystal (Fig. 3). The stacking is mediated by C—H···O inter­actions between ppbp tri­methyl­ene linker C atoms and ppbp amide O atoms (C···O distance = 3.453 (7) Å) and also by C—H···O inter­actions between H_2_oba aromatic C atoms and unprotonated H_2_oba carboxyl­ate O atoms (C···O distance = 3.555 (7) Å).

### S4. Database survey

This compound was not previously reported in the CCDC.

### S5. Synthesis and crystallization

Zinc(II) nitrate hexahydrate and 4,4'-oxydi­benzoic acid (H_2_oba) were obtained commercially. Propane-1,3-diylbis(piperidine-4,1-diyl))bis­(pyridin-4-yl­methanone (ppbp) was prepared *via* modification of a published procedure for the synthesis of piperazine-1,4-diylbis(pyridin-4-yl­methanone) (Hou *et al.*, 2003), using tri­methyl­ene­piperidine instead of piperazine as the amine precursor. A mixture of zinc(II) nitrate hexahydrate (110 mg, 0.37 mmol), H_2_oba (96 mg, 0.37 mmol), ppbp (115 mg, 0.37 mmol), 1.0 mL of a 1.0 *M* NaOH solution, and 10.0 g water (550 mmol) was placed into a 23 ml Teflon-lined Parr acid digestion bomb, which was then heated under autogenous pressure at 393 K for 72 h. Colorless plates of the title compound were obtained along with some amorphous white powder.

### S6. Refinement

All H atoms bound to C atoms were placed in calculated positions, with C—H = 0.95 Å, and refined in riding mode with *U*_iso_ = 1.5*U*_eq_(C). The H atoms bound to O atoms were found in a difference Fourier map, restrained with O—H = 0.85 Å and refined with *U*_iso_ = 1.5*U*_eq_(O).

### Crystal data

**Table:**

C_25_H_32_N_4_O_2_·C_14_H_10_O_5_	*F*(000) = 1440
*M**_r_* = 678.77	*D*_x_ = 1.309 Mg m^−^^3^
Monoclinic, *P*2_1_/*c*	Mo *K*α radiation, λ = 0.71073 Å
*a* = 16.032 (4) Å	Cell parameters from 7245 reflections
*b* = 12.605 (4) Å	θ = 2.4–21.9°
*c* = 17.259 (5) Å	µ = 0.09 mm^−^^1^
β = 99.033 (7)°	*T* = 173 K
*V* = 3444.4 (16) Å^3^	Block, colourless
*Z* = 4	0.22 × 0.17 × 0.15 mm

### Data collection

**Table:**

Bruker APEXII CCD diffractometer	6304 independent reflections
Radiation source: fine-focus sealed tube	3501 reflections with *I* > 2σ(*I*)
Graphite monochromator	*R*_int_ = 0.095
φ and ω scans	θ_max_ = 25.4°, θ_min_ = 2.0°
Absorption correction: multi-scan (*SADABS2012*; Bruker, 2012)	*h* = −19→19
*T*_min_ = 0.715, *T*_max_ = 0.745	*k* = −15→15
55666 measured reflections	*l* = −20→20

### Refinement

**Table:**

Refinement on *F*^2^	Primary atom site location: structure-invariant direct methods
Least-squares matrix: full	Secondary atom site location: difference Fourier map
*R*[*F*^2^ > 2σ(*F*^2^)] = 0.055	Hydrogen site location: inferred from neighbouring sites
*wR*(*F*^2^) = 0.153	H atoms treated by a mixture of independent and constrained refinement
*S* = 1.03	*w* = 1/[σ^2^(*F*_o_^2^) + (0.0533*P*)^2^ + 1.9615*P*] where *P* = (*F*_o_^2^ + 2*F*_c_^2^)/3
6304 reflections	(Δ/σ)_max_ = 0.001
457 parameters	Δρ_max_ = 0.50 e Å^−^^3^
2 restraints	Δρ_min_ = −0.54 e Å^−^^3^

### Special details

**Table:**

Geometry. All e.s.d.'s (except the e.s.d. in the dihedral angle between two l.s. planes) are estimated using the full covariance matrix. The cell e.s.d.'s are taken into account individually in the estimation of e.s.d.'s in distances, angles and torsion angles; correlations between e.s.d.'s in cell parameters are only used when they are defined by crystal symmetry. An approximate (isotropic) treatment of cell e.s.d.'s is used for estimating e.s.d.'s involving l.s. planes.
Refinement. Refinement of *F*^2^ against ALL reflections. The weighted *R*-factor *wR* and goodness of fit *S* are based on *F*^2^, conventional *R*-factors *R* are based on *F*, with *F* set to zero for negative *F*^2^. The threshold expression of *F*^2^ > σ(*F*^2^) is used only for calculating *R*-factors(gt) *etc*. and is not relevant to the choice of reflections for refinement. *R*-factors based on *F*^2^ are statistically about twice as large as those based on *F*, and *R*- factors based on ALL data will be even larger.

### Fractional atomic coordinates and isotropic or equivalent isotropic displacement parameters (Å^2^)

**Table:**

	*x*	*y*	*z*	*U*_iso_*/*U*_eq_	Occ. (<1)
O1	0.64279 (13)	1.41437 (15)	1.60440 (11)	0.0473 (5)	
O2	0.34348 (13)	1.40624 (15)	0.85532 (11)	0.0458 (5)	
O3	−0.00461 (12)	0.80814 (14)	0.23098 (10)	0.0391 (5)	
O4	−0.16756 (13)	1.08325 (16)	−0.06306 (10)	0.0438 (5)	
H4A	−0.1820	1.1041	−0.1095	0.066*	
O5	−0.11387 (14)	0.94796 (16)	−0.12257 (11)	0.0492 (6)	
O6	0.1453 (4)	1.1087 (4)	0.5139 (3)	0.0499 (7)	0.50
O6A	0.1506 (4)	1.0685 (5)	0.5354 (3)	0.0499 (7)	0.50
O7	0.1450 (4)	0.9583 (4)	0.5765 (4)	0.0499 (7)	0.50
O7A	0.1286 (4)	0.9109 (4)	0.5858 (3)	0.0499 (7)	0.50
N1	0.78276 (15)	1.15847 (19)	1.79278 (13)	0.0399 (6)	
N2	0.65133 (14)	1.28497 (17)	1.51423 (12)	0.0338 (5)	
N3	0.32687 (14)	1.28701 (17)	0.95014 (12)	0.0349 (6)	
N4	0.2102 (2)	1.1243 (3)	0.68184 (17)	0.0755 (11)	
C1	0.80512 (17)	1.2576 (2)	1.77677 (15)	0.0379 (7)	
H1	0.8461	1.2932	1.8134	0.045*	
C2	0.77083 (16)	1.3097 (2)	1.70900 (15)	0.0344 (7)	
H2	0.7881	1.3800	1.6997	0.041*	
C3	0.71111 (16)	1.2594 (2)	1.65447 (14)	0.0308 (6)	
C4	0.68972 (18)	1.1559 (2)	1.67015 (15)	0.0357 (7)	
H4	0.6502	1.1177	1.6338	0.043*	
C5	0.72678 (19)	1.1091 (2)	1.73961 (16)	0.0417 (7)	
H5	0.7116	1.0383	1.7499	0.050*	
C6	0.66657 (17)	1.3255 (2)	1.58778 (15)	0.0335 (7)	
C7	0.60681 (18)	1.3526 (2)	1.45183 (15)	0.0407 (7)	
H7A	0.6486	1.3910	1.4258	0.049*	
H7B	0.5727	1.4058	1.4751	0.049*	
C8	0.54927 (17)	1.2874 (2)	1.39107 (15)	0.0356 (7)	
H8A	0.5025	1.2578	1.4156	0.043*	
H8B	0.5243	1.3345	1.3477	0.043*	
C9	0.59577 (17)	1.1970 (2)	1.35774 (15)	0.0327 (6)	
H9	0.6401	1.2291	1.3303	0.039*	
C10	0.64064 (17)	1.1307 (2)	1.42608 (15)	0.0357 (7)	
H10A	0.5979	1.0958	1.4531	0.043*	
H10B	0.6742	1.0745	1.4054	0.043*	
C11	0.69871 (17)	1.1977 (2)	1.48491 (15)	0.0366 (7)	
H11A	0.7243	1.1527	1.5294	0.044*	
H11B	0.7449	1.2272	1.4595	0.044*	
C12	0.53838 (17)	1.1296 (2)	1.29791 (15)	0.0368 (7)	
H12A	0.5730	1.0759	1.2758	0.044*	
H12B	0.4972	1.0915	1.3249	0.044*	
C13	0.49058 (17)	1.1954 (2)	1.23118 (15)	0.0367 (7)	
H13A	0.4503	1.2421	1.2529	0.044*	
H13B	0.5315	1.2416	1.2098	0.044*	
C14	0.44192 (17)	1.1314 (2)	1.16393 (15)	0.0373 (7)	
H14A	0.4081	1.0765	1.1859	0.045*	
H14B	0.4827	1.0946	1.1357	0.045*	
C15	0.37015 (19)	1.3554 (2)	1.01205 (15)	0.0418 (7)	
H15A	0.3279	1.3924	1.0384	0.050*	
H15B	0.4033	1.4096	0.9887	0.050*	
C16	0.42860 (18)	1.2909 (2)	1.07196 (15)	0.0374 (7)	
H16A	0.4536	1.3381	1.1153	0.045*	
H16B	0.4752	1.2621	1.0469	0.045*	
C17	0.38314 (16)	1.1994 (2)	1.10569 (14)	0.0319 (6)	
H17	0.3397	1.2309	1.1346	0.038*	
C18	0.33638 (17)	1.1332 (2)	1.03817 (14)	0.0334 (6)	
H18A	0.3781	1.0969	1.0107	0.040*	
H18B	0.3024	1.0781	1.0597	0.040*	
C19	0.27875 (17)	1.2011 (2)	0.98003 (15)	0.0341 (7)	
H19A	0.2520	1.1565	0.9358	0.041*	
H19B	0.2334	1.2317	1.0060	0.041*	
C20	0.31728 (16)	1.3201 (2)	0.87516 (15)	0.0335 (7)	
C21	0.1847 (2)	1.2234 (4)	0.69088 (19)	0.0684 (12)	
H21	0.1423	1.2519	0.6518	0.082*	
C22	0.21603 (18)	1.2868 (3)	0.75303 (16)	0.0454 (8)	
H22	0.1962	1.3574	0.7561	0.054*	
C23	0.27722 (17)	1.2464 (2)	0.81168 (15)	0.0340 (7)	
C24	0.30416 (18)	1.1427 (2)	0.80403 (16)	0.0409 (7)	
H24	0.3454	1.1116	0.8430	0.049*	
C25	0.2693 (2)	1.0857 (3)	0.7380 (2)	0.0593 (10)	
H25	0.2887	1.0153	0.7324	0.071*	
C26	−0.12708 (16)	0.9924 (2)	−0.06313 (16)	0.0336 (7)	
C27	−0.09764 (16)	0.9483 (2)	0.01669 (14)	0.0294 (6)	
C28	−0.11189 (17)	0.9983 (2)	0.08546 (15)	0.0353 (7)	
H28	−0.1424	1.0631	0.0826	0.042*	
C29	−0.08188 (17)	0.9540 (2)	0.15812 (15)	0.0373 (7)	
H29	−0.0927	0.9875	0.2048	0.045*	
C30	−0.03600 (16)	0.8606 (2)	0.16198 (14)	0.0315 (6)	
C31	−0.02433 (17)	0.8077 (2)	0.09456 (15)	0.0353 (7)	
H31	0.0045	0.7416	0.0976	0.042*	
C32	−0.05520 (17)	0.8522 (2)	0.02257 (15)	0.0347 (7)	
H32	−0.0471	0.8160	−0.0240	0.042*	
C33	0.1311 (5)	1.0135 (7)	0.5137 (4)	0.0499 (7)	0.50
C33A	0.1258 (5)	0.9681 (6)	0.5283 (5)	0.0499 (7)	0.50
C34	0.09603 (18)	0.9448 (3)	0.44368 (16)	0.0453 (8)	
C35	0.09003 (18)	1.0061 (2)	0.37619 (18)	0.0467 (8)	
H35	0.1085	1.0778	0.3799	0.056*	
C36	0.05732 (18)	0.9641 (2)	0.30305 (16)	0.0415 (7)	
H36	0.0546	1.0058	0.2569	0.050*	
C37	0.02892 (16)	0.8607 (2)	0.29897 (14)	0.0314 (6)	
C38	0.03782 (17)	0.7979 (2)	0.36559 (15)	0.0402 (7)	
H38	0.0206	0.7258	0.3618	0.048*	
C39	0.07165 (18)	0.8400 (3)	0.43725 (16)	0.0477 (8)	
H39	0.0782	0.7964	0.4827	0.057*	
H7	0.167 (4)	1.000 (5)	0.614 (3)	0.10 (3)*	0.50
H6A	0.171 (3)	1.088 (5)	0.5825 (19)	0.06 (2)*	0.50

### Atomic displacement parameters (Å^2^)

**Table:**

	*U*^11^	*U*^22^	*U*^33^	*U*^12^	*U*^13^	*U*^23^
O1	0.0639 (14)	0.0333 (12)	0.0412 (12)	0.0047 (10)	−0.0024 (10)	−0.0037 (9)
O2	0.0561 (14)	0.0353 (12)	0.0431 (12)	−0.0068 (10)	−0.0015 (10)	0.0100 (10)
O3	0.0512 (12)	0.0397 (11)	0.0240 (10)	−0.0002 (10)	−0.0021 (9)	0.0004 (9)
O4	0.0481 (13)	0.0512 (13)	0.0307 (11)	0.0085 (11)	0.0016 (10)	0.0056 (10)
O5	0.0685 (15)	0.0533 (13)	0.0243 (11)	0.0073 (11)	0.0031 (10)	−0.0018 (10)
O6	0.0616 (13)	0.051 (3)	0.0325 (16)	−0.0039 (17)	−0.0055 (12)	−0.0009 (16)
O6A	0.0616 (13)	0.051 (3)	0.0325 (16)	−0.0039 (17)	−0.0055 (12)	−0.0009 (16)
O7	0.0616 (13)	0.051 (3)	0.0325 (16)	−0.0039 (17)	−0.0055 (12)	−0.0009 (16)
O7A	0.0616 (13)	0.051 (3)	0.0325 (16)	−0.0039 (17)	−0.0055 (12)	−0.0009 (16)
N1	0.0456 (15)	0.0435 (16)	0.0294 (13)	0.0116 (13)	0.0019 (11)	−0.0008 (11)
N2	0.0411 (14)	0.0305 (13)	0.0271 (12)	0.0045 (11)	−0.0032 (10)	0.0015 (10)
N3	0.0422 (14)	0.0308 (13)	0.0290 (13)	−0.0058 (11)	−0.0031 (10)	−0.0008 (10)
N4	0.051 (2)	0.135 (3)	0.0429 (18)	−0.039 (2)	0.0151 (15)	−0.038 (2)
C1	0.0354 (16)	0.0471 (19)	0.0297 (16)	0.0010 (14)	0.0009 (12)	−0.0081 (14)
C2	0.0351 (16)	0.0354 (16)	0.0322 (15)	−0.0047 (13)	0.0032 (13)	−0.0017 (13)
C3	0.0323 (15)	0.0337 (16)	0.0258 (14)	0.0008 (12)	0.0026 (12)	−0.0002 (12)
C4	0.0449 (17)	0.0327 (16)	0.0280 (15)	0.0005 (13)	0.0013 (13)	−0.0029 (12)
C5	0.055 (2)	0.0330 (16)	0.0366 (17)	0.0022 (15)	0.0065 (15)	−0.0022 (14)
C6	0.0360 (16)	0.0305 (16)	0.0323 (16)	−0.0059 (13)	−0.0004 (12)	−0.0009 (13)
C7	0.0519 (19)	0.0337 (17)	0.0328 (16)	0.0034 (14)	−0.0054 (14)	0.0058 (13)
C8	0.0419 (17)	0.0342 (16)	0.0285 (15)	0.0043 (13)	−0.0008 (12)	0.0036 (12)
C9	0.0338 (15)	0.0363 (16)	0.0274 (14)	−0.0008 (13)	0.0031 (12)	0.0031 (12)
C10	0.0402 (17)	0.0312 (16)	0.0344 (16)	0.0061 (13)	0.0013 (13)	−0.0006 (13)
C11	0.0352 (16)	0.0408 (17)	0.0328 (16)	0.0074 (13)	0.0017 (13)	0.0024 (13)
C12	0.0387 (16)	0.0389 (17)	0.0312 (15)	0.0025 (14)	0.0005 (13)	−0.0017 (13)
C13	0.0356 (16)	0.0419 (17)	0.0315 (15)	0.0003 (14)	0.0018 (12)	0.0008 (14)
C14	0.0383 (17)	0.0400 (17)	0.0324 (16)	−0.0014 (14)	0.0019 (13)	−0.0021 (13)
C15	0.0560 (19)	0.0340 (17)	0.0322 (16)	−0.0095 (15)	−0.0034 (14)	−0.0043 (13)
C16	0.0439 (17)	0.0381 (17)	0.0274 (15)	−0.0083 (14)	−0.0025 (13)	−0.0074 (13)
C17	0.0320 (15)	0.0364 (16)	0.0269 (14)	0.0002 (13)	0.0031 (12)	−0.0014 (12)
C18	0.0365 (16)	0.0329 (16)	0.0298 (15)	−0.0062 (13)	0.0025 (12)	0.0007 (12)
C19	0.0371 (16)	0.0332 (16)	0.0311 (15)	−0.0088 (13)	0.0024 (12)	−0.0001 (12)
C20	0.0309 (15)	0.0336 (17)	0.0344 (16)	0.0029 (13)	0.0000 (12)	0.0019 (13)
C21	0.039 (2)	0.136 (4)	0.0283 (18)	−0.018 (2)	0.0021 (15)	−0.002 (2)
C22	0.0365 (17)	0.069 (2)	0.0292 (16)	−0.0046 (16)	−0.0007 (13)	0.0085 (15)
C23	0.0322 (16)	0.0411 (17)	0.0281 (15)	−0.0035 (13)	0.0032 (12)	0.0038 (13)
C24	0.0440 (18)	0.0410 (18)	0.0380 (17)	−0.0094 (15)	0.0075 (14)	−0.0028 (14)
C25	0.057 (2)	0.064 (2)	0.062 (2)	−0.0238 (19)	0.0262 (19)	−0.0260 (19)
C26	0.0289 (15)	0.0369 (17)	0.0345 (16)	−0.0010 (13)	0.0033 (12)	0.0022 (14)
C27	0.0255 (14)	0.0351 (16)	0.0268 (14)	−0.0057 (12)	0.0014 (11)	−0.0007 (12)
C28	0.0358 (16)	0.0357 (16)	0.0335 (16)	0.0017 (13)	0.0027 (13)	−0.0019 (13)
C29	0.0415 (17)	0.0444 (18)	0.0258 (15)	0.0044 (14)	0.0048 (13)	−0.0038 (13)
C30	0.0322 (15)	0.0369 (16)	0.0239 (14)	−0.0027 (13)	−0.0005 (12)	0.0024 (13)
C31	0.0374 (16)	0.0354 (16)	0.0317 (16)	0.0016 (13)	0.0016 (13)	−0.0038 (13)
C32	0.0386 (16)	0.0401 (17)	0.0245 (14)	0.0010 (14)	0.0019 (12)	−0.0052 (13)
C33	0.0616 (13)	0.051 (3)	0.0325 (16)	−0.0039 (17)	−0.0055 (12)	−0.0009 (16)
C33A	0.0616 (13)	0.051 (3)	0.0325 (16)	−0.0039 (17)	−0.0055 (12)	−0.0009 (16)
C34	0.0309 (17)	0.073 (2)	0.0297 (16)	0.0058 (16)	−0.0011 (13)	−0.0118 (16)
C35	0.0406 (18)	0.0453 (19)	0.051 (2)	−0.0009 (15)	−0.0022 (15)	−0.0143 (16)
C36	0.0456 (18)	0.0470 (19)	0.0303 (16)	−0.0005 (15)	0.0012 (13)	0.0051 (14)
C37	0.0320 (15)	0.0391 (17)	0.0230 (14)	0.0025 (13)	0.0044 (11)	−0.0009 (13)
C38	0.0381 (17)	0.0485 (19)	0.0321 (17)	−0.0032 (14)	−0.0001 (13)	0.0084 (14)
C39	0.0382 (17)	0.075 (2)	0.0278 (16)	−0.0014 (17)	−0.0005 (13)	0.0087 (16)

### Geometric parameters (Å, º)

**Table:**

O1—C6	1.232 (3)	C13—H13A	0.9900
O2—C20	1.232 (3)	C13—H13B	0.9900
O3—C30	1.386 (3)	C13—C14	1.524 (4)
O3—C37	1.381 (3)	C14—H14A	0.9900
O4—H4A	0.8400	C14—H14B	0.9900
O4—C26	1.316 (3)	C14—C17	1.528 (4)
O5—C26	1.217 (3)	C15—H15A	0.9900
O6—C33	1.222 (9)	C15—H15B	0.9900
O6—H6A	1.22 (4)	C15—C16	1.517 (4)
O6A—C33A	1.327 (8)	C16—H16A	0.9900
O6A—H6A	0.86 (2)	C16—H16B	0.9900
O7—C33	1.278 (8)	C16—C17	1.528 (4)
O7—H7	0.86 (2)	C17—H17	1.0000
O7A—C33A	1.221 (9)	C17—C18	1.530 (3)
O7A—H7	1.34 (4)	C18—H18A	0.9900
N1—C1	1.340 (4)	C18—H18B	0.9900
N1—C5	1.332 (3)	C18—C19	1.517 (3)
N2—C6	1.354 (3)	C19—H19A	0.9900
N2—C7	1.468 (3)	C19—H19B	0.9900
N2—C11	1.471 (3)	C20—C23	1.501 (4)
N3—C15	1.460 (3)	C21—H21	0.9500
N3—C19	1.470 (3)	C21—C22	1.368 (5)
N3—C20	1.345 (3)	C22—H22	0.9500
N4—C21	1.331 (5)	C22—C23	1.391 (4)
N4—C25	1.337 (5)	C23—C24	1.390 (4)
C1—H1	0.9500	C24—H24	0.9500
C1—C2	1.378 (4)	C24—C25	1.388 (4)
C2—H2	0.9500	C25—H25	0.9500
C2—C3	1.386 (4)	C26—C27	1.492 (4)
C3—C4	1.386 (4)	C27—C28	1.394 (3)
C3—C6	1.507 (4)	C27—C32	1.386 (4)
C4—H4	0.9500	C28—H28	0.9500
C4—C5	1.384 (4)	C28—C29	1.388 (4)
C5—H5	0.9500	C29—H29	0.9500
C7—H7A	0.9900	C29—C30	1.384 (4)
C7—H7B	0.9900	C30—C31	1.379 (3)
C7—C8	1.524 (4)	C31—H31	0.9500
C8—H8A	0.9900	C31—C32	1.382 (4)
C8—H8B	0.9900	C32—H32	0.9500
C8—C9	1.523 (4)	C33—C34	1.520 (9)
C9—H9	1.0000	C33A—C34	1.493 (9)
C9—C10	1.530 (3)	C34—C35	1.388 (4)
C9—C12	1.528 (4)	C34—C39	1.377 (4)
C10—H10A	0.9900	C35—H35	0.9500
C10—H10B	0.9900	C35—C36	1.393 (4)
C10—C11	1.521 (4)	C36—H36	0.9500
C11—H11A	0.9900	C36—C37	1.379 (4)
C11—H11B	0.9900	C37—C38	1.385 (4)
C12—H12A	0.9900	C38—H38	0.9500
C12—H12B	0.9900	C38—C39	1.377 (4)
C12—C13	1.525 (4)	C39—H39	0.9500
			
C37—O3—C30	122.8 (2)	C17—C16—H16B	109.1
C26—O4—H4A	109.5	C14—C17—C16	113.1 (2)
C33—O6—H6A	80 (3)	C14—C17—H17	107.5
C33A—O6A—H6A	115 (5)	C14—C17—C18	111.9 (2)
C33—O7—H7	107 (6)	C16—C17—H17	107.5
C33A—O7A—H7	76 (3)	C16—C17—C18	109.1 (2)
C5—N1—C1	117.8 (2)	C18—C17—H17	107.5
C6—N2—C7	117.7 (2)	C17—C18—H18A	109.3
C6—N2—C11	125.3 (2)	C17—C18—H18B	109.3
C7—N2—C11	113.5 (2)	H18A—C18—H18B	107.9
C15—N3—C19	113.2 (2)	C19—C18—C17	111.8 (2)
C20—N3—C15	119.3 (2)	C19—C18—H18A	109.3
C20—N3—C19	125.4 (2)	C19—C18—H18B	109.3
C21—N4—C25	116.9 (3)	N3—C19—C18	110.4 (2)
N1—C1—H1	118.8	N3—C19—H19A	109.6
N1—C1—C2	122.3 (3)	N3—C19—H19B	109.6
C2—C1—H1	118.8	C18—C19—H19A	109.6
C1—C2—H2	120.0	C18—C19—H19B	109.6
C1—C2—C3	120.0 (3)	H19A—C19—H19B	108.1
C3—C2—H2	120.0	O2—C20—N3	123.3 (2)
C2—C3—C6	117.4 (2)	O2—C20—C23	117.9 (2)
C4—C3—C2	117.6 (2)	N3—C20—C23	118.8 (2)
C4—C3—C6	124.5 (2)	N4—C21—H21	118.0
C3—C4—H4	120.5	N4—C21—C22	123.9 (4)
C5—C4—C3	119.0 (3)	C22—C21—H21	118.0
C5—C4—H4	120.5	C21—C22—H22	120.4
N1—C5—C4	123.3 (3)	C21—C22—C23	119.2 (3)
N1—C5—H5	118.3	C23—C22—H22	120.4
C4—C5—H5	118.3	C22—C23—C20	118.5 (3)
O1—C6—N2	123.0 (2)	C24—C23—C20	123.2 (2)
O1—C6—C3	116.9 (2)	C24—C23—C22	118.0 (3)
N2—C6—C3	120.1 (2)	C23—C24—H24	120.9
N2—C7—H7A	109.4	C25—C24—C23	118.3 (3)
N2—C7—H7B	109.4	C25—C24—H24	120.9
N2—C7—C8	111.4 (2)	N4—C25—C24	123.7 (3)
H7A—C7—H7B	108.0	N4—C25—H25	118.1
C8—C7—H7A	109.4	C24—C25—H25	118.1
C8—C7—H7B	109.4	O4—C26—C27	114.1 (2)
C7—C8—H8A	109.1	O5—C26—O4	123.6 (3)
C7—C8—H8B	109.1	O5—C26—C27	122.3 (3)
H8A—C8—H8B	107.8	C28—C27—C26	123.2 (2)
C9—C8—C7	112.6 (2)	C32—C27—C26	118.3 (2)
C9—C8—H8A	109.1	C32—C27—C28	118.5 (2)
C9—C8—H8B	109.1	C27—C28—H28	119.7
C8—C9—H9	107.6	C29—C28—C27	120.5 (3)
C8—C9—C10	108.4 (2)	C29—C28—H28	119.7
C8—C9—C12	113.1 (2)	C28—C29—H29	120.3
C10—C9—H9	107.6	C30—C29—C28	119.4 (2)
C12—C9—H9	107.6	C30—C29—H29	120.3
C12—C9—C10	112.2 (2)	C29—C30—O3	124.5 (2)
C9—C10—H10A	109.2	C31—C30—O3	114.5 (2)
C9—C10—H10B	109.2	C31—C30—C29	120.8 (2)
H10A—C10—H10B	107.9	C30—C31—H31	120.5
C11—C10—C9	112.1 (2)	C30—C31—C32	119.1 (3)
C11—C10—H10A	109.2	C32—C31—H31	120.5
C11—C10—H10B	109.2	C27—C32—H32	119.2
N2—C11—C10	110.5 (2)	C31—C32—C27	121.5 (2)
N2—C11—H11A	109.6	C31—C32—H32	119.2
N2—C11—H11B	109.6	O6—C33—O7	121.6 (8)
C10—C11—H11A	109.6	O6—C33—C34	127.4 (6)
C10—C11—H11B	109.6	O6—C33—H6A	50 (2)
H11A—C11—H11B	108.1	O7—C33—C34	111.0 (7)
C9—C12—H12A	109.1	O7—C33—H6A	72 (2)
C9—C12—H12B	109.1	C34—C33—H6A	176 (2)
H12A—C12—H12B	107.8	O6A—C33A—C34	108.9 (6)
C13—C12—C9	112.7 (2)	O6A—C33A—H7	66 (3)
C13—C12—H12A	109.1	O7A—C33A—O6A	121.0 (7)
C13—C12—H12B	109.1	O7A—C33A—C34	130.1 (6)
C12—C13—H13A	108.5	O7A—C33A—H7	56 (3)
C12—C13—H13B	108.5	C34—C33A—H7	173 (3)
H13A—C13—H13B	107.5	C33A—C34—C33	24.4 (3)
C14—C13—C12	115.0 (2)	C35—C34—C33	108.6 (4)
C14—C13—H13A	108.5	C35—C34—C33A	132.9 (4)
C14—C13—H13B	108.5	C39—C34—C33	132.4 (4)
C13—C14—H14A	109.0	C39—C34—C33A	108.1 (4)
C13—C14—H14B	109.0	C39—C34—C35	119.0 (3)
C13—C14—C17	113.1 (2)	C34—C35—H35	119.5
H14A—C14—H14B	107.8	C34—C35—C36	121.0 (3)
C17—C14—H14A	109.0	C36—C35—H35	119.5
C17—C14—H14B	109.0	C35—C36—H36	120.7
N3—C15—H15A	109.5	C37—C36—C35	118.7 (3)
N3—C15—H15B	109.5	C37—C36—H36	120.7
N3—C15—C16	110.8 (2)	O3—C37—C38	114.0 (2)
H15A—C15—H15B	108.1	C36—C37—O3	125.3 (2)
C16—C15—H15A	109.5	C36—C37—C38	120.5 (3)
C16—C15—H15B	109.5	C37—C38—H38	120.0
C15—C16—H16A	109.1	C39—C38—C37	120.0 (3)
C15—C16—H16B	109.1	C39—C38—H38	120.0
C15—C16—C17	112.5 (2)	C34—C39—H39	119.7
H16A—C16—H16B	107.8	C38—C39—C34	120.6 (3)
C17—C16—H16A	109.1	C38—C39—H39	119.7
			
O2—C20—C23—C22	−48.4 (4)	C11—N2—C6—C3	−23.3 (4)
O2—C20—C23—C24	124.8 (3)	C11—N2—C7—C8	54.5 (3)
O3—C30—C31—C32	178.4 (2)	C12—C9—C10—C11	179.2 (2)
O3—C37—C38—C39	179.1 (2)	C12—C13—C14—C17	170.0 (2)
O4—C26—C27—C28	0.3 (4)	C13—C14—C17—C16	55.3 (3)
O4—C26—C27—C32	179.3 (2)	C13—C14—C17—C18	179.0 (2)
O5—C26—C27—C28	179.8 (3)	C14—C17—C18—C19	−179.4 (2)
O5—C26—C27—C32	−1.2 (4)	C15—N3—C19—C18	−57.8 (3)
O6—C33—C34—C33A	−174.6 (18)	C15—N3—C20—O2	0.1 (4)
O6—C33—C34—C35	6.0 (9)	C15—N3—C20—C23	176.5 (2)
O6—C33—C34—C39	−174.2 (6)	C15—C16—C17—C14	177.9 (2)
O6A—C33A—C34—C33	−1.1 (10)	C15—C16—C17—C18	52.7 (3)
O6A—C33A—C34—C35	−0.2 (8)	C16—C17—C18—C19	−53.5 (3)
O6A—C33A—C34—C39	179.2 (5)	C17—C18—C19—N3	56.1 (3)
O7—C33—C34—C33A	5.2 (10)	C19—N3—C15—C16	56.8 (3)
O7—C33—C34—C35	−174.1 (5)	C19—N3—C20—O2	162.5 (3)
O7—C33—C34—C39	5.6 (9)	C19—N3—C20—C23	−21.1 (4)
O7A—C33A—C34—C33	178.0 (19)	C20—N3—C15—C16	−138.8 (3)
O7A—C33A—C34—C35	178.9 (6)	C20—N3—C19—C18	138.8 (3)
O7A—C33A—C34—C39	−1.7 (9)	C20—C23—C24—C25	−172.5 (3)
N1—C1—C2—C3	−0.1 (4)	C21—N4—C25—C24	0.5 (5)
N2—C7—C8—C9	−53.8 (3)	C21—C22—C23—C20	173.9 (3)
N3—C15—C16—C17	−54.4 (3)	C21—C22—C23—C24	0.3 (4)
N3—C20—C23—C22	135.0 (3)	C22—C23—C24—C25	0.8 (4)
N3—C20—C23—C24	−51.7 (4)	C23—C24—C25—N4	−1.2 (5)
N4—C21—C22—C23	−1.0 (5)	C25—N4—C21—C22	0.6 (5)
C1—N1—C5—C4	−1.5 (4)	C26—C27—C28—C29	−179.1 (2)
C1—C2—C3—C4	−1.5 (4)	C26—C27—C32—C31	178.4 (2)
C1—C2—C3—C6	171.1 (2)	C27—C28—C29—C30	1.3 (4)
C2—C3—C4—C5	1.7 (4)	C28—C27—C32—C31	−2.5 (4)
C2—C3—C6—O1	−43.4 (4)	C28—C29—C30—O3	−178.5 (2)
C2—C3—C6—N2	139.2 (3)	C28—C29—C30—C31	−4.1 (4)
C3—C4—C5—N1	−0.2 (4)	C29—C30—C31—C32	3.5 (4)
C4—C3—C6—O1	128.7 (3)	C30—O3—C37—C36	−19.1 (4)
C4—C3—C6—N2	−48.7 (4)	C30—O3—C37—C38	165.1 (2)
C5—N1—C1—C2	1.6 (4)	C30—C31—C32—C27	−0.2 (4)
C6—N2—C7—C8	−145.5 (2)	C32—C27—C28—C29	1.9 (4)
C6—N2—C11—C10	146.0 (3)	C33—C34—C35—C36	−178.1 (4)
C6—C3—C4—C5	−170.3 (3)	C33—C34—C39—C38	177.0 (5)
C7—N2—C6—O1	2.0 (4)	C33A—C34—C35—C36	−178.5 (5)
C7—N2—C6—C3	179.3 (2)	C33A—C34—C39—C38	177.2 (4)
C7—N2—C11—C10	−55.8 (3)	C34—C35—C36—C37	1.6 (4)
C7—C8—C9—C10	53.7 (3)	C35—C34—C39—C38	−3.3 (4)
C7—C8—C9—C12	178.8 (2)	C35—C36—C37—O3	−179.7 (2)
C8—C9—C10—C11	−55.2 (3)	C35—C36—C37—C38	−4.1 (4)
C8—C9—C12—C13	54.6 (3)	C36—C37—C38—C39	3.0 (4)
C9—C10—C11—N2	56.4 (3)	C37—O3—C30—C29	−38.8 (4)
C9—C12—C13—C14	171.8 (2)	C37—O3—C30—C31	146.5 (2)
C10—C9—C12—C13	177.7 (2)	C37—C38—C39—C34	0.7 (4)
C11—N2—C6—O1	159.5 (3)	C39—C34—C35—C36	2.1 (4)

### Hydrogen-bond geometry (Å, º)

**Table:**

*D*—H···*A*	*D*—H	H···*A*	*D*···*A*	*D*—H···*A*
O4—H4*A*···N1^i^	0.84	1.83	2.665 (3)	176
O7—H7···N4	0.86 (2)	2.01 (3)	2.859 (7)	167 (8)
O6*A*—H6*A*···N4	0.86 (2)	1.79 (2)	2.652 (7)	177 (7)
C1—H1···O7*A*^ii^	0.95	2.27	3.114 (6)	147
C12—H12*B*···O2^iii^	0.99	2.60	3.453 (4)	144
C19—H19*B*···O6^iii^	0.99	2.47	3.326 (6)	144

Symmetry codes: (i) *x*−1, *y*, *z*−2; (ii) −*x*+1, *y*+1/2, −*z*+5/2; (iii) *x*, −*y*+5/2, *z*+1/2.
