# Supplementary figures and images for: Crystal structure of [propane-1,3-diylbis(piperidine-4,1-di­yl)]bis­[(pyridin-4-yl)methanone]–4,4′-oxydi­benzoic acid (1/1)
Source: Acta Crystallogr Sect E Struct Rep Online. 2014 Aug 16;70(Pt 9):o1022. doi: 10.1107/S160053681401811X (PMC4186108; doi:10.1107/S160053681401811X)

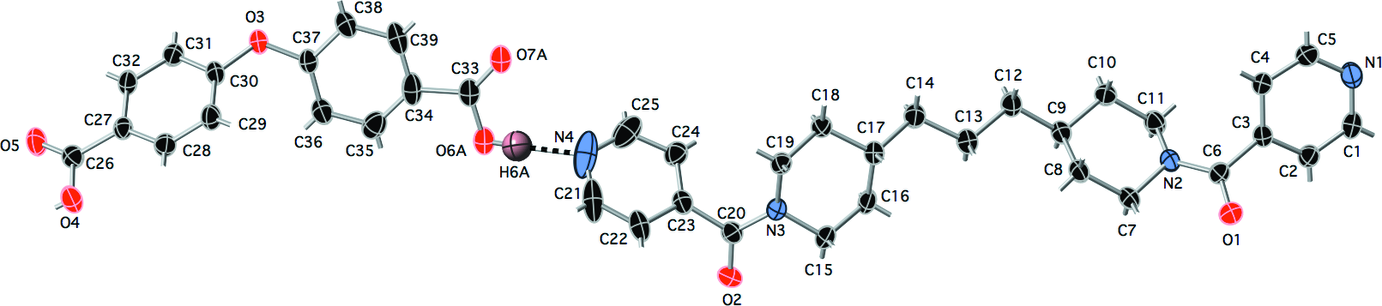

Supplement: Supplementary file 4 [file e-70-o1022-fig1.tif]

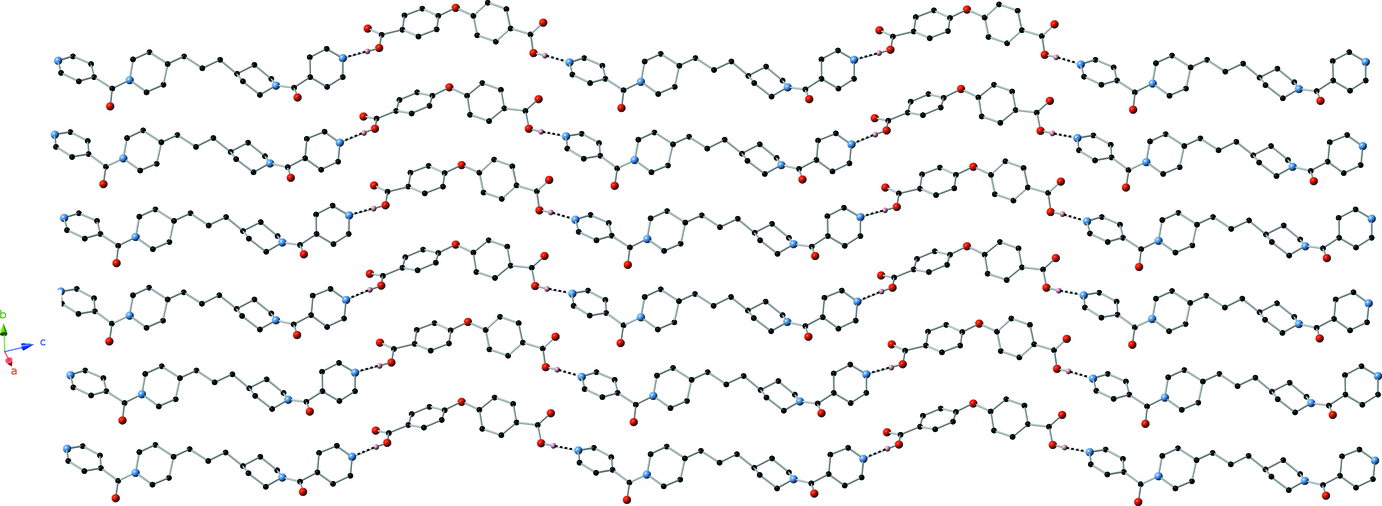

Supplement: Supplementary file 5 [file e-70-o1022-fig2.tif]

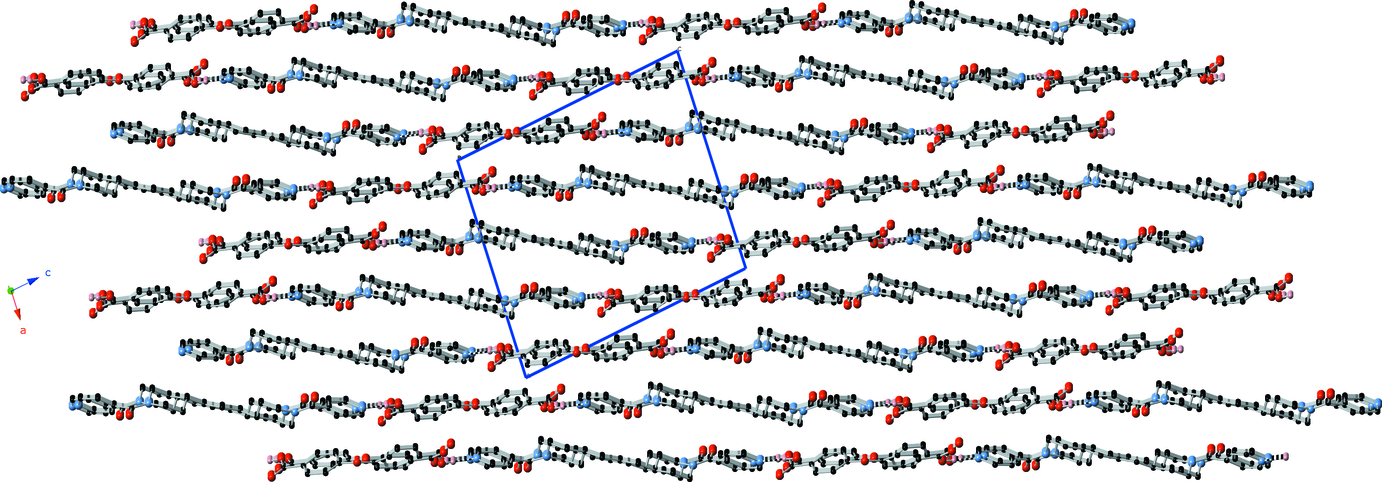

Supplement: Supplementary file 6 [file e-70-o1022-fig3.tif]
